# Effects of age on immune function in broiler chickens

**DOI:** 10.1186/s40104-021-00559-1

**Published:** 2021-03-18

**Authors:** Bochen Song, Dazhi Tang, Shaojia Yan, Hao Fan, Guang Li, Muhammad Suhaib Shahid, Tahir Mahmood, Yuming Guo

**Affiliations:** The State Key Laboratory of Animal Nutrition, College of Animal Science and Technology, China Agricultural University, Beijing, 100193 China

**Keywords:** Age, Broiler chickens, Immunity development

## Abstract

**Background:**

There are many diseases in poultry, many of which are caused by poor immune function. It is not clear how cytokines and various immune cell functions change with age in modern broilers. The purpose of this study was to explore the patterns of development of the immunity of the broiler chickens in cage.

**Results:**

The results showed that there were 3 development patterns of immunity in the broiler chickens. The first pattern was Down-Up. Cytokines and some immune indicators first decreased and then increased, and the lowest levels of immunity basically occurred from d 6 to 13. The second pattern was Up-Down, and from d 30 to 34, the highest levels of non-specific cellular immunity components, such as the peripheral blood mononuclear macrophage ratio, specific cellular immunity components, such as the peripheral blood helper T (Th) cell ratio and T cell and B cell proliferation activity, and mucosal immunity components, such as the ileal *CD4*, *TGF-β1* and *IgA* mRNA levels, were observed. The third pattern was Up-Up, and the levels of the non-specific cellular immunity components, such as the serum nitric oxide (NO), C3 and C4 levels, the specific cellular immunity components, such as the spleen index, peripheral blood IL-2, IFN-γ/IL-4, cytotoxic T (Tc) cell ratio, and splenic *NF-κB* mRNA levels, the humoral immunity components, such as the serum IgG level, the mucosal immunity components, such as the ileal *MHC-II, CD3d, TCRβ subunit, TCRζ subunit, IFN-γ, pIgR* mRNA and ileal mucosa sIgA levels, were continuing to increase from d 1 to 34.

**Conclusions:**

It could be concluded that the immune system and its function have not developed well in the broiler chickens d 6 to 13 and that the immune system does not mature until d 30 to 34 in the broiler chickens in cages. It is necessary to enhance the immune function of the broiler chickens through nutritional measures from d 1 to 30.

**Supplementary Information:**

The online version contains supplementary material available at 10.1186/s40104-021-00559-1.

## Background

The immune organs of birds can be categorized into peripheral immune organs (such as the spleen and caecum tonsil) and central immune organs (such as the thymus, bursa and bone marrow) based on their structures and functions. The central immune organs originate during the embryonic stage and continue to develop from organ primordia to fully functional organs with age. The central immune organs can cultivate mature functional lymphocytes without antigen stimulation and then export these lymphocytes to the peripheral immune system to participate in immune reactions. The development of the peripheral and central immune organs gradually decreases and ceases with sexual maturity [1]. Lymphoid-like stem cells from bone marrow mature in thymus and bursa, and then migrate to peripheral immune organs through blood and lymphatic circulation. These immune cells will proliferate and differentiate when exposed to foreign antigens [2]. Higher proliferative activity in peripheral blood lymphocytes indicated that the activity function of T cells and B cells are getting stronger, also meant that cellular immune function is elevated. The growth and development of immune organs directly determine the overall immune function of birds and affect their resistance to various antigens and stresses in their living environments [3].

Some studies on germ-free animals have shown that symbiotic bacteria, bacterial molecules (such as lipopolysaccharide, β-glucan, and peptidoglycan) and bacterial metabolites (such as retinoid A, SCFA, secondary bile acids, and indole) can fully activate adaptive immune functions [4–9]. The intestinal mucosa plays an important role in the initial activation of the immune response and the subsequent regulation of its maturation [6]. In addition, epidemiological and experimental studies provide increasing evidence that the “growth” of the immune system occurs early in life and depends on the role of the intestinal microbiota [10]. This type of intestinal bacterial ecosystem is disrupted by antibiotics early in the lives of animals and may potentially affect the function of the immune system later in life [10]. Therefore, the immune system can be adjusted during the early growth period by applying immunomodulatory nutritional strategies, such as probiotic additives or directly through nutritional ingredients. Understanding the development of the immune system during chicken growth is a prerequisite for identifying new immunomodulatory factors and strategies.

Maternal antibody plays an important role in protecting chicks against outside challenges especially in the early 3 weeks after hatching. However, maternal antibodies decreased gradually with the increasing age of chickens. On the other hand, part of the live (attenuated) NDV vaccine is neutralized by maternal anti-NDV antibodies. Therefore, the presence of maternal antibodies will interfere with and reduce the immune response of chicks to NDV vaccine during the initial immunization [11]. These factors lead to increased susceptibility of chicks to diseases. Thus, promoting the quick development and maturation of immune system of chickens after hatching is very important to chickens early health. Due to the short feeding period of broilers, the innate immune function is very important for the formation of disease resistance. The application of nutritional interventions can effectively promote the development and maturity of the early immune system of broilers, and enhance the formation of innate immunity of chickens as soon as possible.

Although past studies have also reported on the development of the immune system in broilers [12–15]. There are many existing studies on the number and proportion of immune cells [16, 17] and immunoglobulins [18–22]. Compared with early broilers, due to the fast growth rate, high body weight, high feed conversion rate, short feeding period and high disease susceptibility of modern broilers, these factors require broilers to have strong immune functions. In addition, due to most of the nutrients in the feed are used for muscle growth, fast-growing modern broilers tend to have low disease resistance and high susceptibility to disease. Furthermore, broiler chickens face many threats in the oxygen stress, which will increase disease susceptibility, increase broiler mortality, and affect the healthy growth and economic benefit of broiler chickens. However, the development and maturity of the immune system of modern broilers have not been reported yet. The function of various immune cells (peripheral blood T cell proliferation activity, and B cell proliferative activity), cytokines (IL-2 and IL-10), and immune molecules (NO) during development are relatively poorly understood and require further investigation.

This research on the changes in lymphocytes, cytokines, and gene expression of immune molecules in the spleen and ileum in broilers can help to further understand the immune status of modern broiler chickens at early stages after hatching.

## Methods

### Experimental animals, feeding patterns and diets

A total of 106 male, one-day-old Arbor Acres broiler chickens were selected and reared in laminated cages. The chickens were sacrificed after anaesthesia and sampled on d 1, 6, 13, 20, 27, and 34, with 6 replicates at day 1 and 20 replicates on the subsequent sampling days. According to the recommendation of the NRC (1994), a drug-free, corn-soybean meal diet was prepared to meet or exceed the nutritional requirements of broiler chickens (Table 1). Standard management procedures were used throughout the experiment. Water was supplied through nipple drinkers. Water and feed were provided *ad libitum*.

**Table 1:** Diet composition and nutrient levels

Item, g/kg (unless otherwise indicated)	d 1 to 21	d 22 to 42
Ingredient		
Corn (7.8% CP)	513.8	600.2
Soybean meal (46% CP)	407.1	255.4
Corn protein flour	0.0	56.6
Soybean oil	37.5	33.2
Wheat flour	0.0	20.0
Dicalcium phosphate	18.6	13.3
Limestone	12.4	11.4
Sodium chloride	3.5	3.5
*DL*-Methionine, (98%)	2.0	0.7
*L*-Lys-HCl, (98%)	0.0	1.9
Vitamin premix^a^	0.3	0.3
Mineral premix^b^	2.0	2.0
Choline chloride, (50%)	2.5	1.6
Ethoxyquin, (33%)	0.3	0.0
Nutrient level^c^		
Metabolizable energy, Mcal/kg	2.93	3.10
Crude protein	217.6	200.0
Calcium	10.1	9.0
Available phosphorus	4.4	3.5
Lysine	11.4	10.0
Methionine	5.4	4.0

^a^Supplied per kilogram of complete feed: vitamin A, 12,500 IU; vitamin D_3_, 2,500 IU; vitamin E, 30 IU; vitamin K_3_, 2.65 mg; vitamin B_1_, 2 mg; vitamin B_2_, 6 mg; vitamin B_5_, 12 mg; vitamin B_12_, 0.025 mg; niacin, 50 mg; folic acid, 1.25 mg and biotin, 0.0325 mg

^b^Supplied per kilogram of complete feed: Mn, 100 mg; Fe, 80 mg; Zn, 75 mg; Cu, 8 mg; I, 0.35 mg; and Se, 0.15 mg

^c^Calculated value based on the analysis of experimental diets

### Sampling, determination and immune organ indexes

Six healthy chickens were randomly selected (one for each repeat) on d 1. Twenty healthy chickens were randomly selected for each treatment on d 6, 13, 20, 27, and 34. After weighing, the chickens were euthanized with pentobarbital sodium solution (50 mg/kg body weight) delivered by wing vein injection. The spleen was collected and weighed to determine the immune organ indexes.

Immune organ index (g/kg) = weight of immune organ (g)/live body weight (kg).

### Peripheral blood mononuclear cell isolation

The isolation of peripheral blood mononuclear cells (PBMC) was conducted as previously described [23] using density gradient centrifugation with Ficoll-Paque Plus following the manufacturer’s guidelines. Briefly, Twenty healthy chickens ( bird per replicate) were randomly selected from each treatment group on d 1, 6, 13, 20, 27, and 34. Heparinized blood samples were collected from the wing vein and then diluted 1:1 with sterile calcium- and magnesium-free Hank’s balanced salt solution (CMF-HBSS, Sigma). The diluted samples were placed on ice and then carefully layered into a tube containing an equal volume of Ficoll lymphocyte separation medium (Histopague-1077, Tianjin HaoYang Biological Manufacture Co., Ltd., China) to form a distinct layer above the Ficoll. Following centrifugation at 400×*g* for 30 min at room temperature, the white flocculent material on the interface between the plasma and the lymphocyte separation medium was transferred to a clean tube using a sterile transfer pipette. The lymphocyte suspension was washed 3 times with RPMI 1640 (Invitrogen Corp., Grand Island, NY, USA) incomplete culture medium and then resuspended in 2 mL of RPMI 1640 complete culture medium supplemented with 5% (vol/vol) fetal calf serum, 0.5% penicillin (final concentration, 100 U/mL), 0.5% streptomycin (final concentration, 100 mg/mL), and 1% N-(2-hydroxyethyl)-piperazine-N-2-ethanesulfonic acid (HEPES, final concentration, 24 mmol/L; Amresco 0511, Amresco Inc., Cleveland, OH, USA). The live cells were detected using the Trypan blue dye exclusion technique and a microscope (DM6000B, Leica Microsystems, Wetzla, Germany). The cell suspensions were diluted to a final concentration of 1 × 10^7^ cells/mL in RPMI 1640 medium for subsequent analysis.

### Peripheral blood mononuclear cell proliferation

A 3-(4,5-dimethylthiazol)-2,5-diphenyltetrazolium bromide (MTT, Sigma Chemical Co., St. Louis, MO, USA) assay was used to determine the peripheral blood lymphocyte proliferation response. Briefly, 100 μL of the PBMCs suspension and 100 μL of RPMI 1640 in the absence or presence of 90 μg/mL concanavalin A (Con A; C2613, Sigma Chemical, Co.) or 50 μg/mL lipopolysaccharide (L3129, Sigma Chemical, Co.) were added to a 96-well microtiter plate (Costar 3599, Corning, Inc., Corning, NY, USA). The cultures were set up in triplicate. After a 68-h incubation in a 5% CO_2_ incubator (MCO-18AIC CO_2_ incubator, Sanyo Electric Biomedical Co. Ltd., Tokyo, Japan) at 39 °C, MTT was added to each well at a final concentration of 5 mg/mL. The cells were incubated for an additional 4 h, and then, 100 μL of 10% sodium dodecyl sulfate dissolved in 0.04 mol/L HCl solution was added to each well to lyse the cells and solubilize the MTT crystals. Finally, the absorbance value of each sample was determined using an automated ELISA reader (model 550 Microplate Reader, Bio-Rad Pacific Ltd., Hong Kong, China) at 570 nm. The stimulation index (SI) for each sample was calculated based on the following formula:

SI = (Absorbance value of mitogen - Stimulated cells)/(Absorbance value of media without mitogen).

### Determination of T cell subsets, B cells and monocytes/macrophages in peripheral blood PBMCs by flow cytometry

The percentages of cluster of differentiation 3 receptors CD3+, CD4+, CD8+ and monocyte/macrophage cells in the peripheral blood mononuclear cell samples were analyzed by flow cytometry as previously described [24, 25]. Briefly, the following primary monoclonal antibodies were diluted in PBS (pH 7.2): IgG1κ mouse anti-chicken-CD3-APC-labelled antibody (8200-11), IgG1κ mouse anti-chicken-CD4-Alexa Fluor® 700-labelled antibody (8210-27), IgG1κ mouse anti-chicken-CD8-Pacific Blue™-labelled antibody (8220-26), and IgG1κ mouse anti-chicken-monocyte/macrophage-PE-labelled antibody (8420-09) (Southern Biotechnology Associates Inc., Birmingham, AL). A volume of 100 μL of PBMCs (2 × 10^6^ cells) was added into a 1-mL Eppendorf tube; the contents of the tube were stained with 25 μL of diluted primary monoclonal antibody (1:100 dilution) and the negative isotype control IgG (mouse IgG1κ-APC, mouse IgG1κ-Alexa Fluor® 700, mouse IgG1κ-Pacific Blue™ and mouse IgG1κ-PE). After incubation for 45 min at room temperature, the cells were washed twice with cold PBS and centrifuged for 30 min at 1,800 × *g* to remove the unbound primary antibodies. A total of 300 μL of haemolysin solution diluted in PBS (1:25) was added to each tube. Finally, the cells were washed twice and adjusted to a final volume of 500 μL. Four-colour flow cytometric analysis was conducted using a Navios EX flow cytometer with 10 colors (Beckman Coulter Corp., Fullerton, CA, USA) at Xi-Yuan Traditional Chinese Medicine Hospital, Chinese Academy of Medicine Science, China. The percentages of CD3+ T cells, CD3+CD4+ T cells, CD3+CD8+ T cells and monocytes/macrophages were subsequently calculated.

### Spectrophotometric determination of serum lysozyme activity, NO, complement C3, and C4

On d 1, 6, 13, 20, 27, and 34, blood samples from each treatment group were collected via the wing vein. The levels of lysozyme activity, NO, complement C3, and C4 in the serum samples were measured using commercial methods. The serum lysozyme activity and NO were measured using commercial kits, according to the manufacturer’s instructions (Nanjing Jiancheng Biological Engineering Research Institute Co., Ltd., Nanjing, China). The serum C3 and C4 levels were measured using commercial kits, according to the manufacturer’s instructions (Beijing Leadman Biochemical Co., Ltd., Beijing, China).

### Determination of serum cytokine, immunoglobulin and ileal sIgA levels by ELISA

The levels of IL-1β, IL-2, IL-4, IL-10 and IFN-γ in the serum were determined using commercial ELISA kits (Genorise Scientific Inc., Paoli, USA). Chicken IgG ELISA kit (E30-104, Bethyl Laboratories Inc., Montgomery, TX, USA) was used to determine the level of IgG in the serum according to the instructions. The serum IgA level was determined using a commercial ELISA kit (IDEXX laboratories Inc., Westbrook, Maine, USA) according to the manufacturer’s recommended protocol. The ileal mucosa sIgA level was determined using a commercial ELISA kit (YM-SQ2632, Shanghai yuan Mu Biotechnology Co., Ltd., Shanghai, China).

### Determination of splenic and ileal gene expression by RT-PCR

On d 1, 6, 13, 20, 27, and 34, spleen and ileum molecular samples were quickly frozen in liquid nitrogen and transferred to a − 80 °C freezer to measure the expression of spleen and ileum immune function-related genes. The total RNA of the spleen and ileum were extracted by the TRIzol reagent method (15596018, Invitrogen Life Technologies, Breda, NED). The quality and quantity of the total RNA were measured with a spectrophotometer (NanoDrop-2000, Thermo Fisher Scientific, Waltham, MA, USA) using the 260:280 nm absorbance ratio. First-strand cDNA was synthesized using a PrimeScriptTM RT reagent Kit with gDNA Eraser (Perfect Real Time; Takara Biotechnology Co. Ltd., Dalian, China) according to the manufacturer’s instructions. The cDNA was used to perform quantitative real-time PCR (Applied Biosystems 7500 Fast Real-Time PCR System, USA) for target-gene expression according to the standard protocol [26]. Primer sequences (Table 2) for chicken were designed and synthesized by Sango Biotech Co., Ltd. (Shanghai, China). Using GAPDH as an internal reference, and the results were showed as 2^−△△CT^. The mRNA levels of target genes in the spleen and ileum of 1-day-old broiler chickens were set to 1, to calculate the relative fold changes in the mRNA levels of the same target genes at following day of ages.

**Table 2:** Primers used for real-time PCR analysis^a^

Gene name^b^	Primer sequence (5′ to 3′)	Accession number
*GAPDH*	F:AGAACATCATCCCAGCGTCC	NM_204305
	R:CGGCAGGTCAGGTCAACAAC	
*MHC-II*	F:CCACGGACGTGATGCAGAAC	113,206,149
	R:ACCGCGCAGGAACACGAAGA	
*Mucin 2*	F:TTCATGATGCCTGCTCTTGTG	XM_421035
	R:CCTGAGCCTTGGTACATTCTTGT	
*Lyz C*	F:GACGATGTGAGCTGGCAG	NM_205281
	R:GGATGTTGCACAGGTTCC	
*pIgR*	F:ATTTGTCACCACCACAGCCA	NM_001044644
	R:GAGTAGGCGAGGTCAGCATC	
*IgA*	F:ACCACGGCTCTGACTGTACC	S40610.1
	R:CGATGGTCTCCTTCACATCA	
*NF-κB*	F:TGGAGAAGGCTATGCAGCTT	NM_205134.1
	R:CATCCTGGACAGCAGTGAGA	
*IL-2*	F:AGTGCACCCAGCAAACTCTG	NM_204153.1
	R:TCCGGTGTGATTTAGACCCGT	
*IL-4*	F:GCTCTCAGTGCCGCTGATG	NM_0010079.1
	R:GAAACCTCTCCCTGGATGTCAT	
*IL-6*	F:GATCCGGCAGATGGTGATAA	NM_204628.1
	R:AGGATGAGGTGCATGGTGAT	
*IFN-γ*	F:AAAGCCGCACATCAAACACA	NM_205149.1
	R:GCCATCAGGAAGGTTGTTTTTC	
*TGF-β1*	F:GCCGACACGCAGTACACCAAG	NM_001318456.1
	R:GCAGGCACGGACCACCATATTG	
*CD3d*	F:TGTTGTCGCCACTGTCTTGCTG	NM_205512.1
	R:GTCCATCATTCCGCTCACCAAGG	
*CD4*	F:GATGGAGAGGTGTGGAGCAG	NM_204649
	R:CCTCCTTTCCTGCAATCCCA	
*TCR β subunit*	F:ACTGGTATGCCTGGCCTCTGG	M81149.1
	R:CCACTCCTTCTGTCCTCTTCACAC	
*TCR ζ subunit*	F:AGCTTAGCCAGGCCTCTGAA	AJ002317.1
	R:GGTGCCCAGCACATCGTATT	

^a^Primers designed using Primer Express software (Sangon Biotech, Shanghai, China)

^b^*Abbreviations*: *GADPH* glyceraldehyde-3-phosphate dehydrogenase, *MHC-II* major histocompatibility complex II, *Lyz C* lysozyme C, *pIgR* polymeric immunoglobulin receptor, *IgA* immune globulin A, *NF-κB* nuclear factor kappa-β, *TGF-β1* transforming growth factor-β1, *IL-2* interleukin-2, *IFN-γ* interferon-γ, *TCR β submit* T cell receptor β submit

### Statistical analysis

The experimental data were analyzed using one-way ANOVA with SPSS 20.0 software. The differences between the age groups were analyzed using Duncan multiple comparisons, and the measured indicators were compared with linear and quadratic polynomials. When *P* < 0.05, the difference is significant. The results of this study will be presented in this form: indicator (abscissa of the vertex, R^2^) or indicator (R^2^).

## Results

### The first development pattern: down-up

**Fig. 1:**
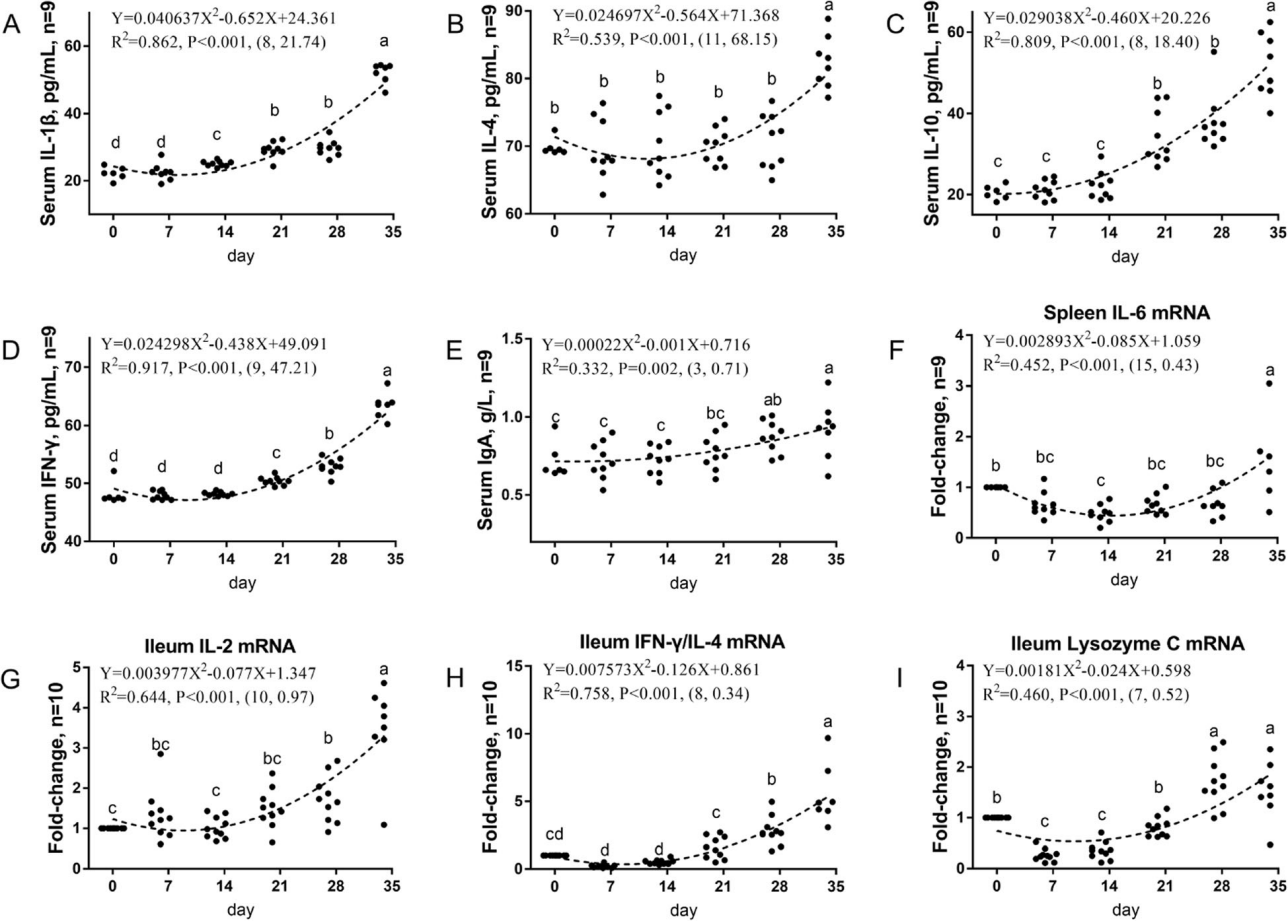
The first immune development pattern in broiler chickens housed in laminated cages is Down-Up. The levels of IL-1β (**a**), IL-4 (**b**), IL-10 (**c**), IFN-γ (**d**) and IgA (**e**) in serum were analyzed by ELISA kit. The mRNA levels of *IL-6* in spleen (**f**), *IL-2* (**g**), *IFN-γ/IL-4* (**h**) and *Lysozyme C* (**i**) in ileum were analyzed by RT-PCR. Different small letters indicated significant difference (*P* < 0.05)

The first developmental pattern was Down-Up, and it was shown in Table 3 and Fig. 1. Immune function first decreased to a certain age; the lowest point of immune function basically occurred from d 6 to 13 and then, immune function continued to increase until d 34. Most of the indicators assessed were serum cytokines, including IL-1β (8, 0.862), IL-4 (11, 0.539), IL-10 (8, 0.809), IFN-γ (9, 0.917), IgA (3, 0.332) levels in the serum, *IL-6* (15, 0.452) mRNA level in the spleen, and *IL-2* (10, 0.644), *IFN-γ/IL-4* (8, 0.758), *Lysozyme C* (7, 0.460), and *NF-κB* (19, 1.59) mRNA levels in the ileum.

**Table 3:** Down-Up: immunity indicators that decrease first and then increase in broiler chickens from d 1 to 34

Immunity indicators	Vertex coordinates^a^	R^2^	Regression equation
Serum IL-1β, pg/mL	(8, 21.74)	0.862	Y = 0.040637X^2^–0.652X + 24.361
Serum IL-4, pg/mL	(11, 68.15)	0.539	Y = 0.024697X^2^–0.564X + 71.368
Serum IL-10, pg/mL	(8, 18.40)	0.809	Y = 0.029038X^2^–0.460X + 20.226
Serum IFN-γ, pg/mL	(9, 47.21)	0.917	Y = 0.024298X^2^–0.438X + 49.091
Serum IgA, g/L	(3, 0.71)	0.332	Y = 0.00022X^2^–0.001X + 0.716
Spleen *IL-6*	(15, 0.43)	0.452	Y = 0.002893X^2^–0.085X + 1.059
Ileum *IL-2*	(10, 0.97)	0.644	Y = 0.003977X^2^–0.077X + 1.347
Ileum *IFN-γ/IL-4*	(8, 0.34)	0.758	Y = 0.007573X^2^–0.126X + 0.861
Ileum *Lysozyme C*	(7, 0.52)	0.46	Y = 0.00181X^2^–0.024X + 0.598

^a^The X and Y of the vertex coordinates are the age and extreme value respectively

### The second developmental pattern: up-down

**Fig. 2:**
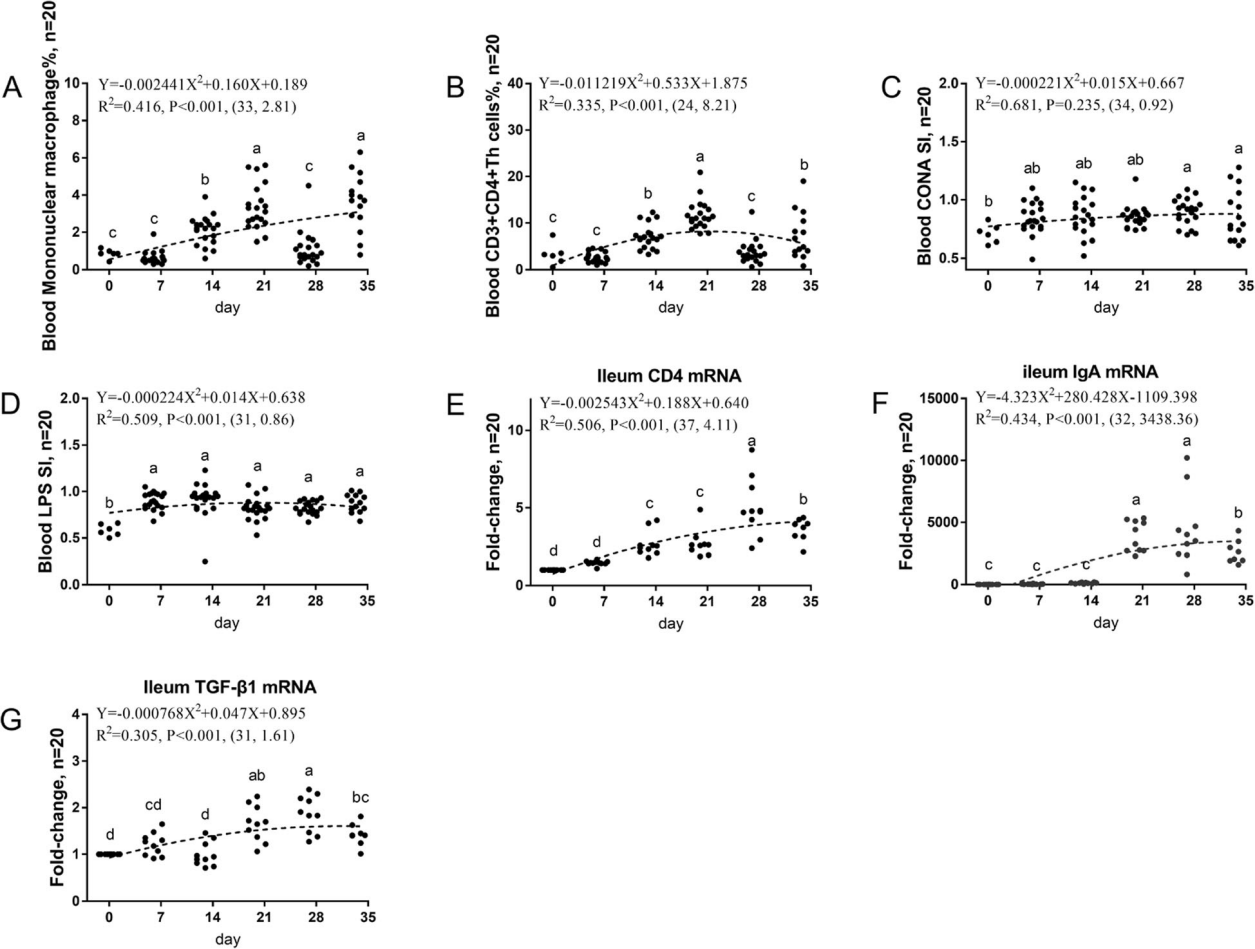
The second immune development pattern of broiler chickens housed in laminated cages is Up-Down. The frequencies of mononuclear/macrophage (**a**) and Th cells (**b**) of peripheral blood lymphocytes were analyzed by flow cytometry. Peripheral blood lymphocytes were stimulated with concanavalin A (ConA) (**c**) or lipopolysaccharide (LPS) (**d**), and the stimulation index (SI) was calculated as described in the Materials and Methods section. The mRNA levels of *CD4* (**e**), *IgA* (**f**) and *TGF-β1* (**g**) were analysed by RT-PCR. Different small letters on the bars indicated significant difference (*P* < 0.05)

The second developmental pattern was Up-Down, and it was shown in Table 4 and Fig. 2. The highest point in immune function basically occurred within d 30 to 34. Several changes in immune function conformed to this pattern, including the proportion of mononuclear macrophages (33, 0.416), the Th cell ratio (24, 0.355), the T cell proliferation activity (34, 0.681), the B cell proliferation activity (32, 0.509) in peripheral blood, and the *CD4* (37, 0.506), *IgA* (32, 0.434), and *TGF-β1* (31, 0.305) mRNA levels in the ileum.

**Table 4:** Up-Down: immunity indicators that increase first and then decrease in broiler chickens from d 1 to 34

Immunity indicators	Vertex coordinates^a^	R^2^	Regression equation
Mononuclear macrophage ratio	(33, 2.81)	0.416	Y = −0.002441X^2^ + 0.160X + 0.189
Peripheral blood CD3+CD4+ Th cell ratio	(24, 8.21)	0.355	Y = −0.011219X^2^ + 0.533X + 1.875
Peripheral blood CD3+ T cell proliferation activity	(34, 0.92)	0.681	Y = −0.000221X^2^ + 0.015X + 0.667
Peripheral blood B cell proliferation activity	(32, 0.86)	0.509	Y = −0.000224X^2^ + 0.014X + 0.638
Ileum *Mucin 2*	(19, 2.65)	0.462	Y = −0.004697X^2^ + 0.183X + 0.869
Ileum *CD4*	(37, 4.11)	0.506	Y = −0.002543X^2^ + 0.188X + 0.640
Ileum *TGF-β1*	(31, 1.61)	0.305	Y = −0.000768X^2^ + 0.047X + 0.895
Ileum *IgA*	(32, 3438.36)	0.434	Y = −4.323X^2^ + 280.428X–1109.398

^a^The X and Y of the vertex coordinates are the age and extreme value respectively

### The third developmental pattern: up-up

**Fig. 3:**
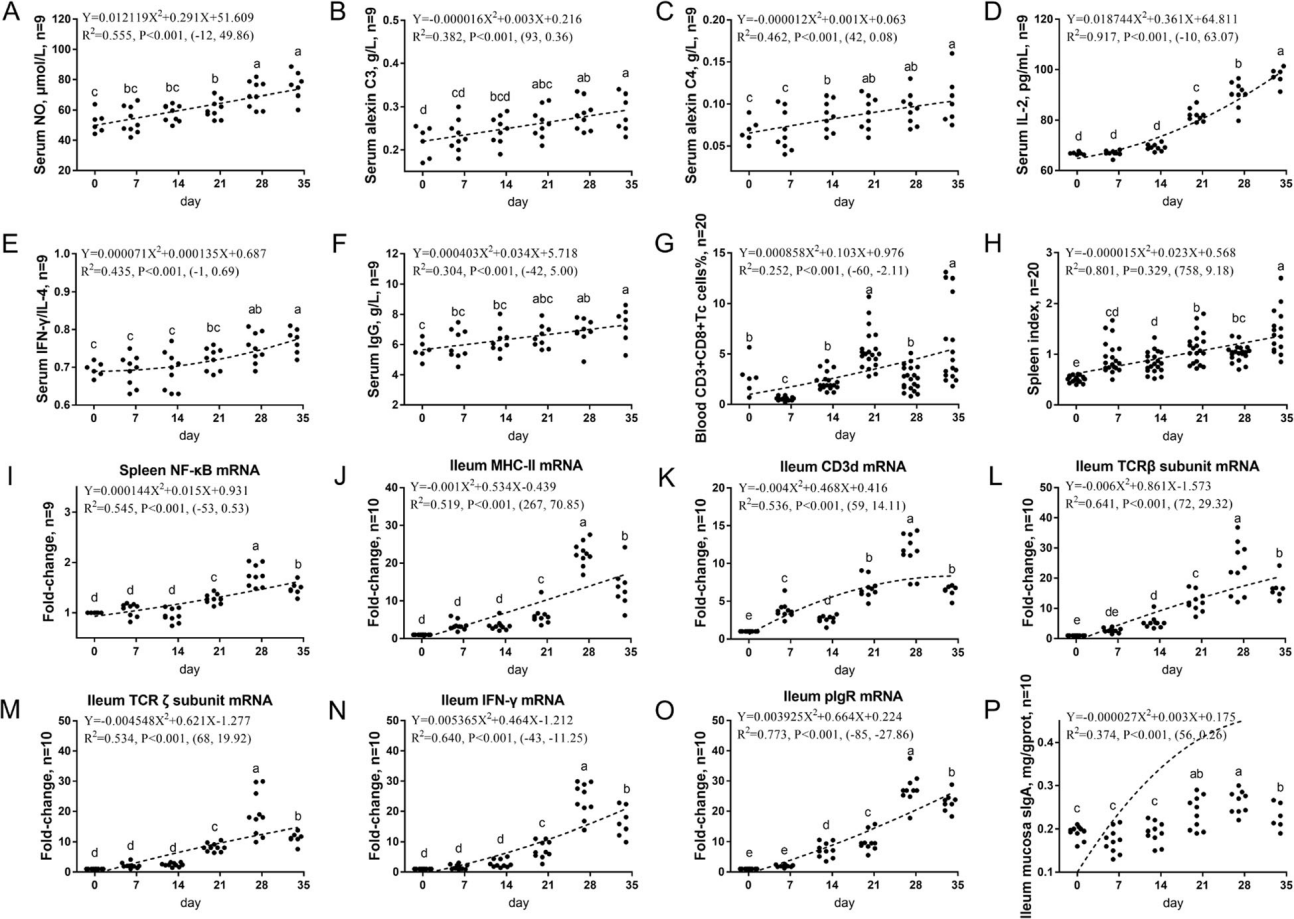
The third immune development pattern of broiler chickens housed in laminated cages is Up-Up. The levels of NO (**a**), C3 (**b**), C4 (**c**), IL-2 (**d**), IFN-γ/IL-4 (**e**), IgG (**f**) in serum and sIgA in ileum mucosa (**p**) were analyzed by ELISA kit. The frequencies of Tc cells (**g**) of peripheral blood lymphocytes were analyzed by flow cytometry. The spleen index was analyzed by weighing (**h**). The mRNA levels of *NF-κB* in spleen (**i**), *MHC-II* (**j**), *CD3d* (**k**), *TCRβ subunit* (**l**), *TCRζ subunit* (**m**), *IFN-γ* (**n**) and *pIgR* (**o**) in ileum were analyzed by RT-PCR. Different small letters indicated significant difference (*P* < 0.05)

The third developmental pattern was Up-Up, and it was shown in Table 5 and Fig. 3. The indicators of this pattern were mainly indicators of peripheral blood non-specific immunity, mucosal cell immunity and humoral immunity, including the spleen index (0.801) and the NO (0.555), C3 (0.382), C4 (0.462), IL-2 (0.917), IFN-γ/IL-4 (0.435), and IgG (0.304) levels, the Tc cell ratio (0.252) in the peripheral blood, the NF-κB (0.545) mRNA level in the spleen, the MHC-II (0.519), CD3d (0.536), TCRβ subunit (0.641), TCRζ subunit (0.534), IFN-γ (0.640), and pIgR (0.773) mRNA levels in the ileum, and the sIgA level in the ileal mucosa (0.374).

**Table 5:** Up-Up: continuously rising immune indicators in broiler chickens from d 1 to 34

Immunity indicators	Vertex coordinates^a^	R^2^	Regression equation
Serum NO, μmol/L	(−12, 49.86)	0.555	Y = 0.012119X^2^ + 0.291X + 51.609
Serum C3, g/L	(94, 0.36)	0.382	Y = −0.000016X^2^ + 0.003X + 0.216
Serum C4, g/L	(42, 0.08)	0.462	Y = −0.000012X^2^0.001X + 0.063
Serum IL-2, pg/mL	(−10, 63.07)	0.917	Y = 0.018744X^2^ + 0.361X + 64.811
Serum IFN-γ/IL-4	(−1, 0.69)	0.435	Y = 0.000071X^2^ + 0.000135X + 0.687
Peripheral blood CD3+CD8+ Tc cell ratio	(−60, −2.11)	0.252	Y = 0.000858X^2^ + 0.103X + 0.976
Serum IgG, g/L	(−42, 5.00)	0.304	Y = 0.000403X^2^ + 0.034X + 5.718
Spleen index	(758, 9.18)	0.801	Y = –0.000015X^2^ + 0.023X + 0.568
Spleen *NF-κB*	(−53, 0.53)	0.545	Y = 0.000144X^2^ + 0.015X + 0.931
Ileum *MHC-II*	(267, 70.85)	0.519	Y = −0.001X^2^ + 0.534X-0.439
Ileum *CD3d*	(59, 14.11)	0.536	Y = −0.004X^2^ + 0.468X + 0.416
Ileum *TCRβ subunit*	(72, 29.32)	0.641	Y = −0.006X^2^ + 0.861X−1.573
Ileum *TCRζ subunit*	(68, 19.92)	0.534	Y = −0.004548X^2^ + 0.621X−1.277
Ileum *IFN-γ*	(−43, −11.25)	0.64	Y = 0.005365X^2^ + 0.464X−1.212
Ileum *pIgR*	(−85, −27.86)	0.773	Y = 0.003925X^2^ + 0.664X + 0.224
Ileal mucosa sIgA, ng/mg	(56, 0.26)	0.374	Y = −0.000027X^2^ + 0.003X + 0.175

^a^The X and Y of the vertex coordinates are the age and extreme value respectively

## Discussion

The first development pattern was Down-Up, i.e. the IL-1β, IL-4, IL-10, IFN-γ levels in the peripheral blood, the IL-2 gene expression in the spleen, the IL-2, IFN-γ/IL-4, and Lysozyme C gene expression in the ileum first decreased and then increased, and the lowest levels of immunity basically occurred from d 6 to 13, and the main indicators of this pattern were cytokines. This implies that the peripheral cellular immune function in broiler chickens did not develop well from d 6 to 13. Within d 6 to 13, the immune system has not yet matured, and the chick cannot produce enough cytokines. Low levels of cytokines may slow down the differentiation of stem cells into mature immune cells and lead to poor disease resistance in broilers. Cytokines are polypeptides or glycoproteins that are synthesized and secreted mainly by various immune cells. Cytokines can mediate the interaction between cells and have a variety of biological functions, such as regulating cell growth, differentiation and maturation, regulating the immune response, and participating in inflammation and wound healing. Studies have shown that IL-4 exhibits dual immunomodulatory activities [27]. The lysozyme gene is gradually activated in mature macrophages and is constitutively expressed at high levels [28].

Previous studies have shown that from d 1 to 7, the expression of the IL-β gene in the duodenum and caecum of broiler chickens reached its highest level on d 2, but this expression continued to increase in the colorectal region [29]. The research by Lowenthal showed that when splenic T cells isolated from d 1 to 7 chickens were stimulated by ConA, the IL-2 and IFN levels produced by these cells continued to increase as aged [12]. It is also reported that the mRNA expression levels of IL-1, IL-10, IL-12p40, iNOS, and IFN-γ in the jejunum and ileum of d 14 to 42 chickens are higher than those in d 3 and d 49 to 70 chickens (*P* < 0.05) [12], and this is in line with our current work. Our study shown that after the lowest point of 6 to 13 d, the peripheral blood cytokine levels of broiler chickens continued to increase.

The second developmental pattern was Up-Down, i.e. the mononuclear macrophage proportion, T cell proliferation activity and B cell proliferation activity of broiler peripheral blood cells reached the highest points at d 30 to 34. Mononuclear macrophages belong to the mononuclear phagocytic system, and considered to be the first line of immune defence and represent an important step of interactions with infectious agents. As mobile scavenger cells, macrophages participate in innate immunity by acting as phagocytes. Macrophages use their abilities as antigen-processing and antigen-presenting cells to initiate an “acquired” immune response. In response to their tissue microenvironment or to foreign antigens, macrophages may secrete several immunomodulatory cytokines or metabolites [30]. The lymphocyte proliferation test is a routine experimental method for assessing the cellular immune function of the body. Lymphocytes and mitogens are transformed into primitive lymphoblasts when they are cocultured *in vitro*, and characteristic morphological changes occur. The degree of mitogen-induced lymphocyte proliferation is speculated to reflect the lymphocyte functional status *in vivo*. The ileal mucosal tissue contains mucosal lymphoid tissue and produces IgA to prevent invasion by pathogens. CD4 is mainly expressed by Th cells, and it is the receptor through which Th cells recognize the TCR antigen, and the ratio of Th cells is an important indicator of the body’s immunity. TGF-β mainly plays roles in inflammation, tissue repair, and embryonic development and has important regulatory effects on the immune function, growth and differentiation of cells [31]. TGF-β expressed in the thymus may have the ability to regulate immature thymic cells by accelerating the cell cycle and their differentiation into CD3-positive thymic cells [32].

The ileal IgA gene expression reached its highest point at d 32, and this finding may be different from the continuous increasing trend of the ileal mucosa sIgA level due to the effects of posttranscriptional and posttranslational regulation. Overall, the ileal mucosal humoral immune function of broiler chickens between d 30 and d 42 was relatively mature. The percentage of Th cells in the peripheral blood and CD4 gene expression in the ileum reached their highest points at approximately d 30, which are indicators of the peripheral and mucosal T cell immunity development. The immune regulator TGF-β1, which mainly plays an anti-inflammatory role, also reached its highest point at d 30.

Previous studies have shown that from d 1 to 7, the phagocytic index of *Salmonella typhimurium* in d 4 chickens did not change significantly compared with that in d 1 chickens but significantly increased in d 7 chickens [33]. By examining all data from time points, the highest phagocytic capacity was observed at d 31 in the present work. The reason for the different results of the activity measurements may be the differences in the test animal species, test methods and measurement time points. The ratios of CD4+CD8− cells in the thymus and CD4−CD8+ and CD4+CD8+ cells in the spleen of 7-week-old broiler chickens are higher than those of 2-week-old broiler chickens, and this may indicate a stronger immune system in the chickens aged 7 weeks [16]. This study shown that the T cell immune development in the peripheral immune organs of broiler chickens was stronger at approximately d 35, and these results were similar to those found in our study at d 24 (peripheral blood Th cell ratio) and 36 (ileal CD4 gene expression). Studies have shown that the proportions of CD3-, CD4-, CD8-, γδ-TCR-, αβ-Vβ1-TCR-, and αβ-Vβ2-TCR-positive cells in the thymus, spleen, and blind flat of Lai Hen laying hens reached their highest points at week 15 [17]. Our study shown that the percentage of Th cells in the peripheral blood and CD4 gene expression in the ileum reached their highest points at approximately d 30. The reason for the different time points may be the type of chicken. The study shown that the IgA gene expression in the ileum and caecum of broiler chickens and Peking ducks was low at d 1 to 7, rapidly increased at d 7 to 14, and was stably expressed after reaching the highest point at d 21 [22]. These findings are similar to our study, but the reason for the difference between d 21 and d 32 may be because our study assessed immunity of broiler chickens in six times points, so the time points derived from the regression equation may be more accurate. Studies have shown that in the jejunum and ileum of laying chickens, IgM gene expression peaks at week 1, IgY gene expression peaks at week 5, and IgA gene expression continues to increase [20]. The reason for the difference between this study and our study in the IgA patterns may be the different breeds of poultry. The study shown that in d 12 to 17 chicken embryo chondrocytes, the expression of TGF-β2 and TGF-β4 mRNA increased with age, whereas the expression of TGF-β3 mRNA was not related to age. In cardiomyocytes cultured from d 7 to 9 chicken embryo hearts, TGF-β2, TGF-β3, and TGF-β4 mRNA expression is constitutive [34]. In immune cells, TGF-β regulates the production and function of many types of immune cells and is an important promoter of immune homeostasis and immune tolerance, inhibiting the expansion and function of various components of the immune system. Our study shown that TGF-β1 expression in the ileum reached its highest point at d 30, along with other immune function indicators, which may indicate that the peripheral and mucosal T cell immune function of the body matures at approximately d 30. In addition, to prevent the body from entering a state of excessive immune function and to maintain the body’s immune balance, the immune regulator TGF-β1, which mainly plays roles in maintaining stability and inhibiting inflammation, also reached its highest point at d 30. The reason for the difference from the previously reported patterns may be that the TGF-β subtype and the site assessed are different.

The third developmental pattern was Up-Up. The peripheral blood and mucosal non-specific immunity (peripheral blood NO, C3, and C4 and ileal MHC-II), peripheral and mucosal cell immunity (peripheral blood IL-2 levels and Tc cell ratio and ileal CD3d, TCRβ subunit, TCRζ subunit, and IFN-γ gene expression), Th1/Th2 balance (peripheral blood IFN-γ/IL-4 levels), peripheral and mucosal humoral immunity (peripheral blood IgG levels, ileal pIgR gene expression, and ileal mucosa sIgA levels), and the immune organ development of the spleen (spleen index and NF-κB gene expression) continued to develop from d 1 to 34. NO is an important component of non-specific immunity. The PAMPs of macrophages stimulated by bacterial LPS and IFN-γ produce NO. NO has immunomodulatory properties, such as regulating NF-κB-dependent signal transduction pathways, IκB activity and the expression of various cytokines, etc., and it’s usually used as a marker of innate immunity [35, 36]. In the current research, the serum NO level of broiler chickens was in a state of continuous development from d 1 to 34. Complement is mainly synthesized by macrophages and the liver, can be used as an intermediary between antibodies and phagocytes, and is a key component that can enhance humoral and cell-mediated specific immunity [37]. It’s reported that the serum complement levels in chickens from week 5 to 6 reach the level observed in adult chickens [38]. This finding was similar to our results that the serum C3 and C4 levels of broiler chickens was in a state of continuous development from d 1 to 34. Dendritic cells are the most powerful professional antigen-presenting cells in the body. Dendritic cells can efficiently ingest, process, and present antigens. Mature DCs can effectively activate initial T cells and are at the centre of the initiation, regulation, and maintenance of immune responses. The expression of cell surface markers, such as MHC-II, CD40, CD80 and CD86, is considered to be a sign of the maturation of dendritic cells in mammals [39]. Serum IgG, IgM and IgA contents are humoral immune molecules produced by active humoral immune cells in the body’s immune organs and tissues and are important indicators of the functional status of the humoral immune system [40]. sIgA prevents pathogenic bacteria from adsorbing to and entering epithelial cells, protecting intestinal epithelial cells from intestinal toxins and pathogenic microorganisms [41]. pIgR is a key component of sIgA. pIgR transports polymeric IgA from the lamina propria to luminal mucin and is expressed on the basal lateral surface of the liver, bursal bladder and intestinal epithelium [21]. The development of the avian immune system is mainly characterized by the balanced regulation of the Th1/Th2 immune response capacity. Helper T cells (Th) are mainly divided into two subpopulations, namely, Th1 and Th2 cells, according to their secretion of cytokines. Th1 cells mainly secrete IFN-γ, IL-2 and TNF-α, which can downregulate IgA secretion, activate macrophages, enhance cytotoxicity, and mediate cellular immune response [42]; Th2 cells mainly secrete IL-4, IL-6 and IL-10, which can stimulate B cell proliferation and antibody secretion, enhance secreted IgA gene expression, and promote humoral immune responses [43]. The Th1/Th2 balance is the main mechanism by which the immune stability of the body is maintained. The Th2 immune response predominates during incubation. After incubation, stimulated by external antigens (especially microbial antigens), the Th1 immune response continues to increase, Th1/Th2 functions tend to be balanced, and immune function gradually matures.

Studies have shown that MHC-II+ -like cells are observed in the intestine of chicken embryos at embryonic day 13, and then, Bu-1+ cells and IgM+ cells are observed; at the same site where MHC-II+ -like cells are found, the number of Bu-1+ and IgM+ cells is increased, and similar results have been obtained in the caecum tonsils [44]. Our work showed that MHC-II and IgG gene expression in the ileum continued to increase from d 1 to 34. MHC-II is a surface marker of mature DC cells, indicating that ileal non-specific immunity continues to develop from d 1 to 34.

The expression of the CD3d, TCRβ subunit and TCRζ subunit genes in the ileum of broiler chickens continued to increase, indicating that the immune system of broiler intestinal mucosa T cells is continuously developing from d 1 to 34.

IL-2 is mainly secreted by activated CD4+ T cells. Our study shown that the levels of IL-2 in the peripheral blood continue to increase, which means that the number of mature CD4+ T cells gradually increases. In addition, the serum IFN-γ/IL-4 levels and ileal IFN-r gene expression also continued to increase from d 1 to 34, which means that the Th1 immune response of chickens was continuously enhanced, Th1/Th2 cells tend to be balanced, and the immune function gradually matures.

It is reported that the number of IgG-binding cells increases with embryonic age and is maintained a high level in the bursa of chicken embryos after incubation; a peak is observed at embryonic d 16 in the thymus [45]. In this work, the serum IgG levels in broiler chickens continued to increase, which may be related to the continuous increase in the number of IgG-binding cells in the bursa and thymus observed in previous studies. The research by Lammers et al. showed that in the jejunum and ileum of laying chickens, IgM gene expression peaks at week 1, IgY gene expression peaks at week 5, and IgA gene expression continues to increase [20]. The study by Zhang et al. found that in the ileum and caecum of broiler chickens and Peking ducks, IgA gene expression was low from d 1 to 7 and then rapidly increased from d 7 to 14, reaching the highest point at d 21; pIgR gene expression continued to increase after hatching [22]. We found that similar previous findings, the broiler ileal IgG and pIgR gene expression and mucosal sIgA levels continued to increase from d 1 to 34, indicating that broiler mucosal humoral immunity is continuously developing.

## Conclusion

It could be concluded that the immune system and its function did not develop well from d 6 to 13. The main manifestations are the low peripheral blood cytokine levels and intestinal mucosa cytokine expression within d 6 to 13.

The immune system did not mature until d 30 to 34. The main indication was that peripheral blood cellular immune function reaches its strongest level within d 30 to 34.

Many immune indicators continue to increase from d 1 to 34, indicating that during this period, the immune system of broiler chickens is still developing. The main manifestations are that the indicators of non-specific cellular immunity, specific cellular immunity, specific humoral immunity in the peripheral blood and mucosal immunity continue to increase from d 1 to 34.

## Supplementary Information

**Additional file 1. Figure S1.** The flow cytometry and flow histograms of lymphocytes in the peripheral blood of broiler chickens in 1, 6, 13, 20, 27 and 34 day of ages.

## Data Availability

The datasets produced and/or analyzed during the current study are available from the corresponding author on reasonable request.

## Abbreviations

C3: Complement 3
CD3d: Cluster of differentiation 3d
Con A: Concanavalin A
ELISA: Enzyme-linked immuno sorbent assay
IFN-γ: Interferon-γ
IL-1β: Interleukin-1β
LPS: Lipopolysaccharide
Lyz: Lysozyme
MHC-II: Major histocompatibility complex-II
MTT: 3-(4,5-Dimethylthiazol)-2,5-diphenyltetrazolium bromide
NF-κB: Nuclear factor-κB
NO: Nitrogen oxide
PBMC: Peripheral blood mononuclear cell
pIgR: Polymeric immunoglobulin receptor
RT-PCR: Reverse transcription-rolymerase chain reaction
SI: Stimulation index
sIgA: Secretory immunoglobulin A
Tc cell: Cytotoxic T cell
TCRβ subunit: T cell receptor β subunit
TGF-β1: Transforming growth factor-β 1
Th cell: Helper T cell
