# Supplementary material for: Effects of age on immune function in broiler chickens
Source: J Anim Sci Biotechnol. 2021 Mar 18;12:42. doi: 10.1186/s40104-021-00559-1 (PMC7971956; doi:10.1186/s40104-021-00559-1)
Supplement: Supplementary file 1 — Additional file 1. Figure S1. The flow cytometry and flow histograms of lymphocytes in the peripheral blood of broiler chickens in 1, 6, 13, 20, 27 and 34 day of ages. [file 40104_2021_559_MOESM1_ESM.docx]

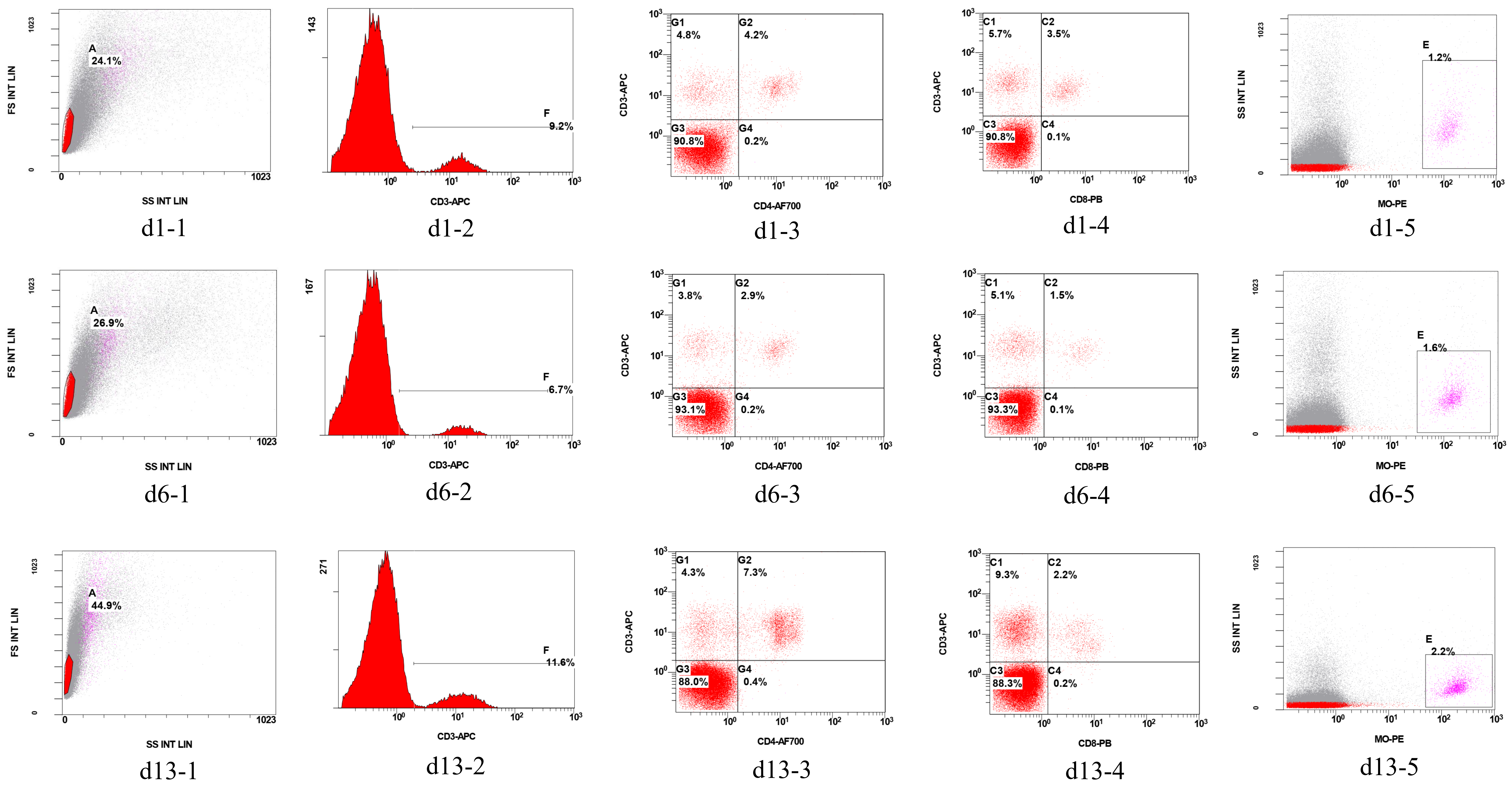


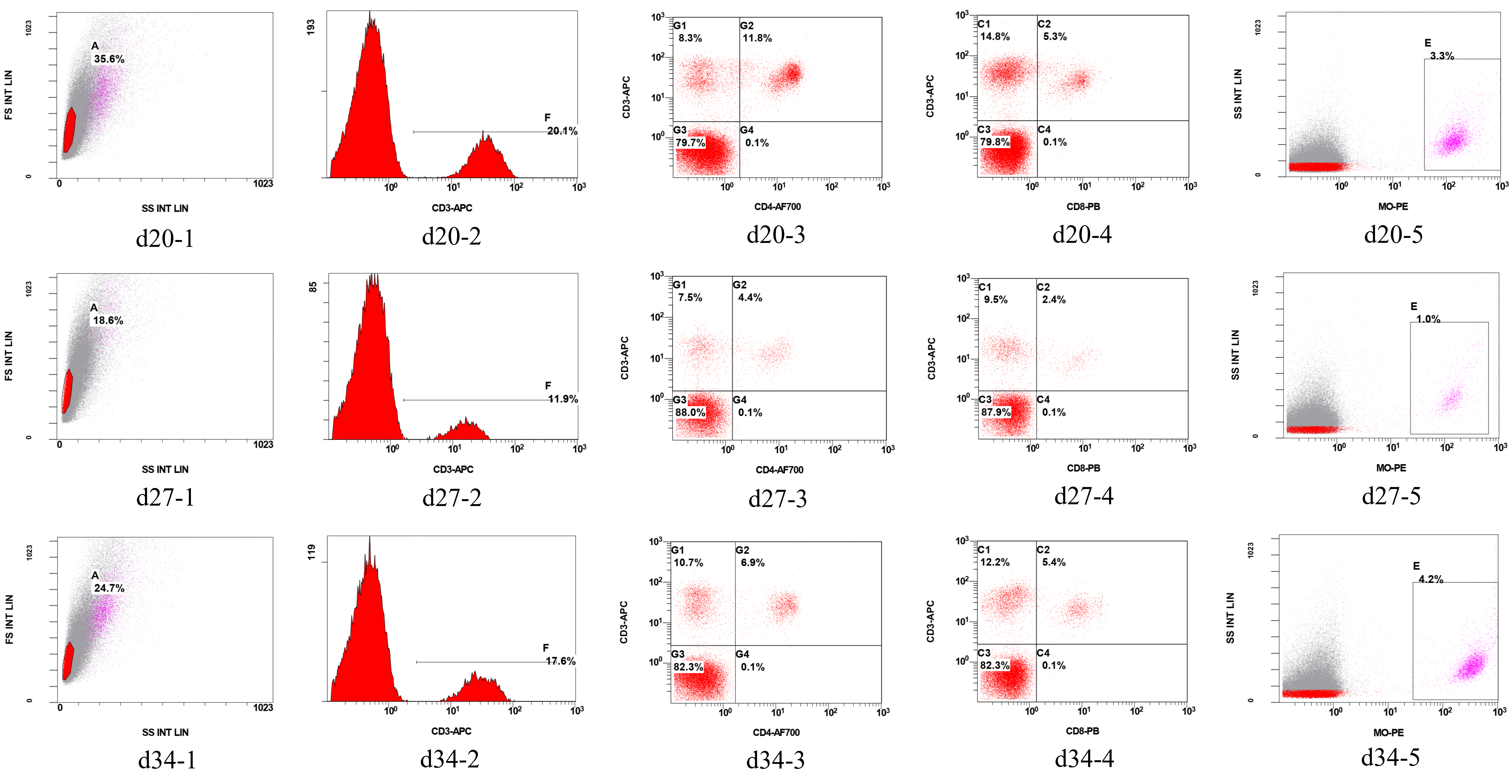


Figure S1. The flow cytometry and flow histograms of lymphocytes in the peripheral blood of broiler chickens in 1, 6, 13, 20, 27 and 34 day of ages.
